# Long-Term Follow-Up Results of Delayed Fixation of Femoral Neck Fractures in Adults

**DOI:** 10.5812/traumamon.11275

**Published:** 2013-05-26

**Authors:** Asghar Elmi, Ali Tabrizi, Alireza Rouhani, Fardin Mirzatolouei

**Affiliations:** 1 Department of Orthopedics and Trauma Surgery, Shohada Educational Hospital, Tabriz University of Medical Sciences, Tabriz, IR Iran; 2 Department of Orthopedic Surgery, Motahari Hospital, Urumieh University of Medical Sciences, Urumieh, IR Iran

**Keywords:** Femoral Neck Fractures, Fracture Fixation, Internal, Bone Screws

## Abstract

**Background:**

Femoral neck fractures are urgent injuries that require precise reduction and stable fixation. In some cases, however, early treatment is not possible.

**Objectives:**

The present study aimed to evaluate long-term results of delayed fixation of femoral neck fractures using cannulated screws.

**Patients and Methods:**

This retrospective descriptive-analytical study was conducted on 26 patients with femoral neck fractures. The patients were treated through a closed reduction and fixation method using cannulated screws. Patients were followed up for at least five years and the rate of complications was determined.

**Results:**

In this study, 26 patients with mean age of 34.3 years were assessed. Average time interval from injury to surgery was 46.4 ± 12.2 hours; 18 patients (69%) were operated on with more than 36 hours of delay. Incidence of AVN and nonunion was reported in 10 (38.4%) and 3 (11.5%) patients, respectively.

**Conclusions:**

Time plays an important role in treatment results of femoral neck fractures. To treat the fractures, closed reduction and fixation using cannulated screws may still be the best option.

## 1. Background

Intra-capsular femoral neck fractures are treated as an emergency in adults (younger than 60 years old) and quick efforts should be made to restore blood supply of the femoral head (1). Some believe that such cases should be regarded as “vascular emergencies” and operative interventions should be rendered within 6-8 hours (1, 2). Two major complications threatening these patients include nonunion and avascular necrosis (AVN) of the femoral head (3, 4). Incidence of nonunion and aseptic necrosis of the femoral neck following osteosynthesis is estimated at 10-30% and 15-30%, respectively (3, 4). The main purpose of treatment is to restore function to the pre-injury state. However, most studies suggest that early fixation with proper reduction is necessary for good results. In some cases, the problem is not diagnosed and patients with delayed fractures are referred to orthopedic centers (4). In adults and adolescents, femoral neck fractures usually occur due to high-energy traumas and are accompanied by several multi-system injuries. Therefore, so much time is spent on examining other organs that the golden time is lost for early surgical treatment of femoral neck fractures (5, 6). Although femoral neck fractures usually occur due to high-energy trauma, they may also result from low-energy trauma (5, 7). 

There are some differences between young adults and older patients considering treatment of femoral neck fractures. According to the literature, closed reduction with internal fixation (CRIF) is the best treatment method since it is effective, less invasive, and requires less operation time (5). Treatment and fear of complications in patients with delayed femoral fractures are important concerns of orthopedics surgeons.

## 2. Objectives

The present study aimed to assess long-term results of delayed fixation of femoral neck fractures using cannulated screws.

## 3. Patients and Methods

In this descriptive-analytical study conducted from 2005 to 2012, 26 patients (younger than 60 years) with displaced intra-capsular fractures of femoral neck (Garden grades 1 and 2) were evaluated. The patients were referred to our center and evaluated after 12 hours. Patients with multi-organ injuries, multiple fractures, history of previous femoral fracture or hip surgery, systemic diseases or metabolic disorders like diabetes or cardiovascular diseases, and lower limb deformities were not assessed. Patients were followed for at least 5 years. The study was confirmed by the Ethics Committee of Tabriz University of Medical Sciences.

### 3.1. Operation Technique

The patients were evaluated on a traction table; Minimal traction and rotation was applied first. Reduction was controlled by using a C-arm on both anterior-posterior (AP) and lateral views. The junction point of the convex femoral head and neck had to produce an S-shaped curve in all planes. A Steinmann pin was inserted into the distal femur in order to provide better control during manipulation and traction. Fracture reduction and fixation was achieved using three cannulated screws. The screws were placed through the lateral approach placed parallel or convergent to each other in a large triangular configuration using an angle guide. All screws were placed a minimum angle of 130° to the shaft of the femur. The first and second screws were respectively placed along the calcar and posterior cortex while the third one was placed at the superior part of the neck. When closed reduction failed, open reduction was performed in some patients. The Watson-Jones approach was used in these patients and the fracture was opened using an inverted T-shaped incision in the capsule. 

### 3.2. Follow-Up

On the first day postoperatively the patients were seated at the edge of a bed and shown quadriceps isometric exercises. During the second day, with the nurse’s assistance, they walked with a pair of crutches (non-weight bearing) around the room. Patients without any other problems were discharged three days after operation. To rehabilitate the patients, they were allowed active assisted mobilization of the hip as soon as pain was mitigated and began touch weight-bearing at six weeks. Partial weight-bearing was allowed after 12 weeks. Additionally, full weight-bearing was permitted when there was clinical and radiological evidence of union. The patients were followed up for at least five years. Nonunion was defined as clinical signs of instability at the fractured area or pain in the hip requiring further surgery, persistence of or an increase in the fracture gap, sclerosis of margins of the fracture, change of the screws orientation in relation to the bone or change of orientation of two fractured fragments. AVN was defined as appearance of subchondral sclerosis or presence of segmental collapse.

### 3.3. Statistical Analysis

Descriptive statistical method (frequency, percentage), mean ± SD, and Medcalc software were used to statistically analyze the data.

## 4. Results

**Figure 1.**
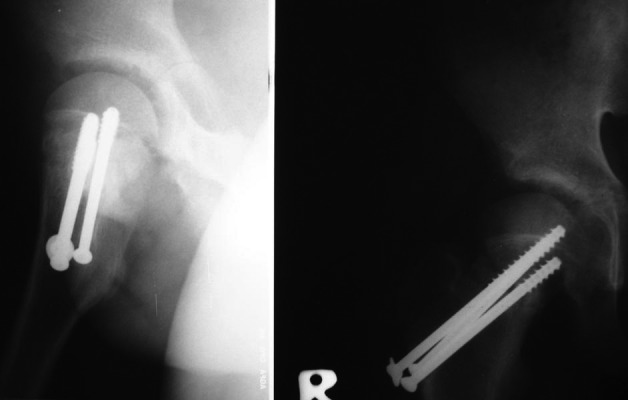
One Month After a Closed Reduction and Internal Fixation in a 16 Year-Old Patient Treated After 36 Hours

**Figure 2.**
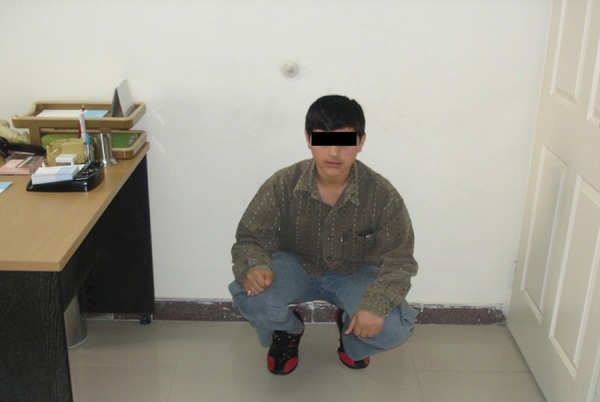
Sitting Ability of the Same Patient One Year After Treatment

**Figure 3.**
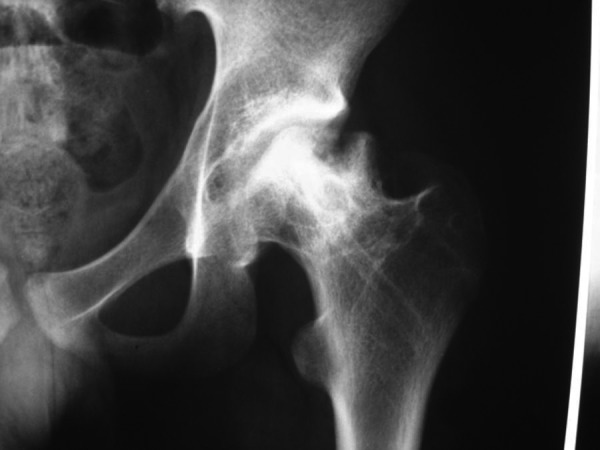
Radiography of the Hip in the Same Patient After Three Years and Removal of Screws

Of the 26 patients included in the study, 21 (80.7%) were men and 5 (19.2%) women. Car accidents or falls were found to be the main cause of femoral neck fractures. Average time interval from injury to surgery was 46.4 ± 12.2 hours and 18 patients (69%) were operated with more than 36 hours of delay. The patients were operated once it was administratively possible after admission. According to preoperative radiographs, grades 1 and 2 were observed in 16 (61.5%) and 10 (38.4%) patients respectively. In this study, 5 (19.2%) patients were treated using the open reduction method and the rest underwent the closed reduction method. AVN of the femoral head was observed in those patients treated with open reduction after a 36-hour delay. There were no differences in the frequency of symptoms between the two grades. There were 2 complicated patients (40% AVN) who were treated with open reduction. Table 1 demonstrates patients’ characteristics and their follow-up results. Incidence of AVN and nonunion were reported in 10 (38.4%) and 3 (11.5%) patients, respectively. Figures 1, 2 and 3 show a young patient treated with approximately 48-hours of delay. 

**Table 1.** Patients Characteristics and Results of Follow up

Variable	n = 26
**Sex, Male/Female, %**	80.7/19.2
**Age, mean ± SD, y** ^**a**^	34.3 ± 12.5
**Time Before Surgery, mean ± SD, h**	46.4 ± 12.2
**Delay More Than 36 h**	18 (69)
**Time to Union, mean ± SD,wk**	16.8 ± 4.02
**Time of Follow up, mean ± SD, y**	5.6 ± 1.2
**Open Reduction, No. (%)**	5 (19.2)
**Closed Reduction, No. (%)**	21 (80.8)
**Nonunion, No. (%)**	3 (11.5)
**AVN, No. (%)**	10 (38.4)
**Infection in Surgery Site, No. (%)**	1 (3.8)
**DVT, No. (%)**	2 (7.6)

^a^ Abbreviations: AVN, avascular necrosis; DVT, deep venous thrombosis; h, hour; wk, week; y, year

## 5. Discussion

Due to the complex anatomy of the femoral neck and its unique blood supply, early treatment and fixation is of high importance (8). Treatment goals for young patients suffering from femoral neck fractures include, improvement of fracture outcome through preserving the femoral head, prevention of non-union and avascular necrosis, and return of patients to their previous functional status with a quick rehabilitation (6, 8). Fracture stability is important considering selection of treatment modality. Garden fractures I&II are defined as stable and III&IV are unstable fractures (6, 8). Nowadays, fixation of femoral neck fractures using three cannulated screws is preferred since it leads to adequate stabilization (9). It is recommended that internal fixation should be done within the first 24 hours post-injury. However, some studies suggest a fixation time of less than 8 hours especially in dislocated fractures (6). In younger patients, even a very trivial delay is important. Therefore, femoral neck fractures are considered as a true orthopedic emergency in young patients (6). According to the literature, early reduction and fixation of femoral neck fractures in young adults leads to ideal results (9). Swiontkowski et al. reported low rates of AVN (20%) and no symptomatic nonunions in young patients. This success may have been attributed to the application of an institutional protocol of immediate reduction (within eight hours of diagnosis) and internal fixation with compression (3). Other studies referred to the correlation found between time interval of surgery and nonunion and AVN. In their studies Gerber et al.(10) and Robinson et al.(11) similarly indicated low rates of AVN and nonunion in early-treated patients. According to Jain et al.(12), AVN rate was 16 % for patients treated more than 12 hours after injury. In different studies, nonunion varies from 4% to 59% and AVN from 10% to 86% (13). This significant variation may be attributed to differences in selection of patients considering their age, bone quality, fracture pattern, reduction method, fixation mode, and surgery time (7, 14). According to previous reports, incidence of AVN following neglected femoral neck fractures varies from 0% to 67%. However, this is below 15% in most reports (1). The relationship between osteonecrosis and non-union is not fully understood (1). In their study, Upadhyay et al.(7) suggested that more-than-48-hours of delay before surgery did not influence the rate of union or development of AVN when compared with operations within 48 hours of injury (7). Smektala et al. demonstrated that time-to-surgery does not affect mortality (15). According to their study, shorter time-to-surgery may be associated with lower rates of some complications (decubitus ulcers, urinary tract infections, thromboses, pneumonia and cardiovascular events) with higher rates of other (postoperative bleeding or implant) complications (15). Asnis and Wanek-Sgaglione (16) demonstrated that AVN increased from 11% in two years to 22% in eight years. Non-displaced fractures of Garden grade I and II were seen in about 25% of their patients. According to our study, incidence rate of AVN was 38.4% and nonunion rate was slightly higher than that of the previous studies. Although early treatment may prevent serious complications, there are apparently other associated factors affecting results of treatment and complications. However, it seems that fixation using cannulated screws is the best treatment option (6). Time has an important role in patient outcomes but it seems that it is not the only influential factor. Factors such as the patient's bone quality, reduction status, metabolic and nutritional status may affect treatment results in delayed femoral neck fractures. Further studies are recommended to evaluate these factors. There is no difference between our study and previously conducted research considering nonunion. Upadhyay et al.(7) studied 102 patients with femoral neck fractures. The patients were treated tardily and nonunion was seen in 18.7% of their patients. In our study, nonunion was observed only in 11% and time interval between trauma and surgery had no effect on union. Closed reduction and fixation with cannulated screws may still be the best treatment option in patients with femoral neck fractures.
